# Aerosol morphology and particle size distribution in orthopaedic bone machining: a laboratory worst-case contamination simulation. Is high-speed bone machining potentially harmful by pollution and quality schemes and what measures could be taken for prevention?

**DOI:** 10.1007/s00264-022-05398-x

**Published:** 2022-04-18

**Authors:** David Putzer, Dietmar Dammerer, Cora Huber, Harald Boschert, Martin Thaler, Michael Nogler

**Affiliations:** 1 Department of Orthopaedics and Traumatology—Experimental Orthopaedics, Medical University of Innsbruck, Sonnenburgstrasse 16, 6020 Innsbruck, Austria; 2 Department of Orthopaedics and Traumatology, Medical University of Innsbruck, Innrain 36, 6020 Innsbruck, Austria; 3 Stryker Leibinger GmbH & Co. KG, Bötzinger Strasse 41, 79111 Freiburg, Germany; 4 Helios Klinikum, Arthroplasty Center Munich West, Steinerweg 5, 81241 Munich, Germany

**Keywords:** High-speed bur, Bone machining, Size distribution of aerosol particles, Morphology of aerosol particles, Contamination by aerosols

## Abstract

**Aim of the study:**

High-speed bone machining devices with irrigation fluid were used in surgery to spread aerosols and toss tissue particles of varying morphology into the operating room. Based on measurements taken on a phantom object, the shape, size, and spatial contamination distribution of such particles were assessed.

**Method:**

Cadaveric femoral heads were continuously machined with a spherical bur, manually held at a fixed attack angle. The irrigation fluid used during bone machining was enriched with bacteria to act as a tracer to quantify the spatial contamination. A vertical board equipped with snippets served as a phantom object to assess contamination load and morphology of airborne particles.

**Results:**

Eight-nine percent of the particles had a non-circular cross section. The detected particle size ranged across six orders of magnitude, from 0.006 to 4 mm2 with a median particle size of 0.125 mm2. The CFU counts observed after the standard machining time ranged from 7 to 240, with a median of 2 CFUs. The highest median contamination was seen at the upper right corner of the phantom.

**Discussion:**

The experiments show that contaminating particles of a wide variety of shapes and sizes are part of the aerosol created by high-speed burring. While protection of personnel and equipment is always important, surgical helmets should be worn, especially at contamination hotspots, and gloves should be replaced at the end of machining. Sensitive instruments and measuring devices—such as optical sensors—should also be protected effectively, as the optical measurement may be obstructed by aerosol particles.

## Introduction

High-speed burs are being used in robotic assisted surgery [1–4] as well as in conventional surgery [5–7] for shaping bones. High-speed burs heat up by friction which can induce bone necrosis and protein denaturation. The degree of necrosis depends strongly on the bur type, machining time and speed, and the force introduced by the operator [8–12]. To prevent osteonecrosis irrigation is essential as a coolant to reduce temperature when exposing bone [8, 13, 14]. However, when high-speed machining devices are used in combination with irrigation, aerosols are produced and tissue particles are tossed into the theater [15–17].

Being aware of hot spots of contamination and the distribution of airborne spread during aerosol-generating medical procedures in the operation room (OR) is crucial [15–20]. These aerosols are not only a threat for medical professionals during their daily work in the OR, but they also create a risk of contamination of the surrounding area including sterile equipment and materials [17, 19, 20]. Schultz et al. reported a contamination rate with patients’ dermal bacteria of 35% during bone resection and only 10% in the control group, where no bone machining occurred [21]. Besides causing surgical site infection [17], a high risk of transmitting virus particles such as SARS-CoV-2 is known to be present in all body fluids and in tissue particles [22, 23].

Aerosols can contain both fluid and solid particles [24]. They can be categorized into three groups based on their aerodynamic diameter [25]:

1. Small particles of aerodynamic diameters between 5 μm and 10 μm: They follow airflow streamlines and are responsible for short- and long-range transmission.
2. Intermediate particles of aerodynamic diameters between 10 μm and 20 μm: They combine properties of small and large droplets, but settle more quickly than particles smaller than 10 μm, and potentially carry a smaller infectious dose than droplets larger than 20 μm.
3. Large particles of aerodynamic diameters beyond 20 μm: They follow a more ballistic trajectory. These droplets are too large to follow airflow streamlines; surgical masks can be considered an effective barrier for them.

Based on the aerodynamic diameter, particles smaller than 100 μm are affecting the upper respiratory tract, particles with a diameter smaller than 25 μm can reach the lower respiratory tract, and particles smaller than 10 μm can reach the alveoli.

Surgical masks, a personal protective equipment, can prevent the motion of airborne particles between patients and health care personal by acting as a barrier to block the transmission of pathogens. However, several studies showed that the efficacy of filtering airborne particles depends on their particle size [26, 27].

The aim of this study was to assess the morphology and size distribution as well as the spatial contamination by aerosols, by emulating an aerosol generating orthopedic procedure in a laboratory environment.

## Materials and methods

### Experimental setup

**Fig. 1:**
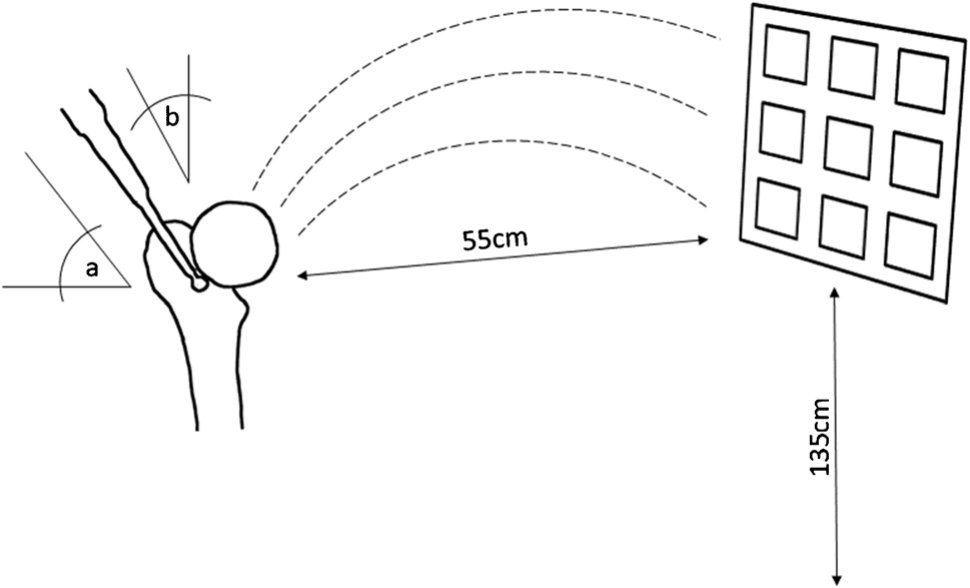
Schematic illustration of the arrangement of snippets on the phantom object. Positions A, B, C, and D were used for contamination load analysis and positions 1, 2, 3, and 4 for the morphology analysis

**Fig. 2:**
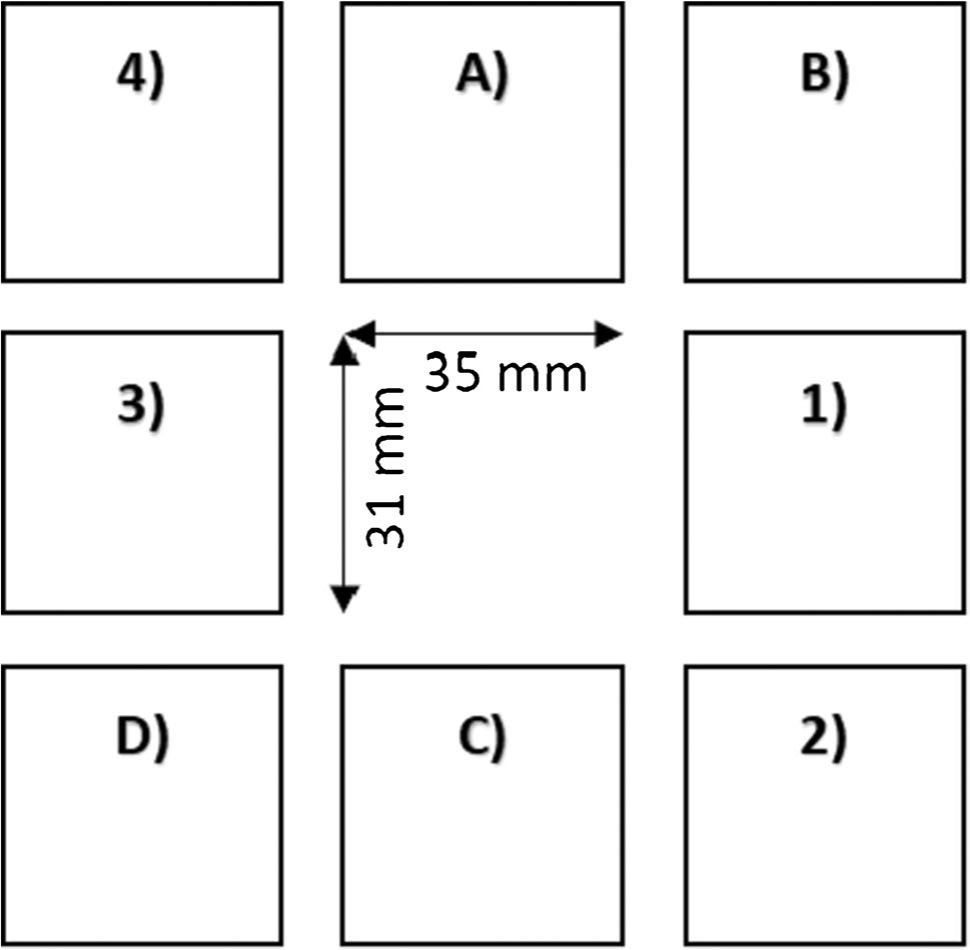
Schematic illustration of the attack angles of the cutting device applied in this assessment. The operator moved the burr on the bone surface with the angle *α* = 70° in the sagittal view and with the angle *β* = 65° in the frontal view

The contamination load and particle morphology were assessed in a laboratory environment. To create a worst-case scenario of maximum contamination, a phantom object was placed at a previously determined “hot spot” in 55-cm distance to the machining site, 135 cm above the floor [18] (see Fig. 1). The phantom object, essentially a vertical board, was equipped with eight snippets, sized 35 mm × 31 mm (3 M anti-fog hydrophilic polyester film, thickness 0.1 mm; part number 9962) with a tape, serving as contamination recipients for the aerosol particles (Fig. 2). A vertical board was chosen to investigate contamination levels on screens, navigation cameras, arthroscopy towers, and other medical devices positioned nearby the bone resection spot. To verify that the phantom snippets to be used had not been contaminated prior to the test, four of them were arbitrarily selected and submitted to direct contact testing.

In addition to the eight phantom snippets, four room reference snippets (“RR”) per test were placed vertically in the room for compensation of a potential contamination of the room air. Room reference snippet “RR1” was placed at a height of 0.55 m in the proximity of the irrigation pump about 1.5 m off the burring spot to detect any contamination caused by connecting the irrigation fluid to the pump. Room reference snippet “RR2” was placed at a height of 1.5 m behind the phantom object, 4 m off the burring spot. Room reference snippet “RR3” and “RR4” were placed at a height of 1.5 m on the left and right side of the phantom object at a minimum distance of 2 m off the burring spot.

Attention was paid to not pre-contaminate the snippets with particles or contaminated solution, fingerprints, etc., during preparation. To emulate a worst-case scenario, no laminar flow system was provided. For the same purpose, no additional staff members or equipment—which could potentially block the spreading of aerosols—were placed in the simulated OR environment. The room temperature was 22 °C; room humidity was not controlled and not measured.

Non-embalmed cadaveric human femoral heads were rigidly mounted on a corkscrew. All donors had given their informed consent for donation. Pursuing the worst-case approach, no soft tissue envelope was present which could limit the spreading of particles. The femoral heads were machined with a manually guided high-speed cutting device. A πDrive® Drill (Stryker Instruments, Kalamazoo, MI, USA) was equipped with a 7-cm Elite Straight Attachment and a 6-mm round bur driven at 40,000 rpm.

(Elite Round Fluted Zyphr; Stryker Instruments, Kalamazoo, MI, USA).

The initial attack angles of the bur were 70° in the sagittal view and 65° in the frontal view as illustrated in Fig. 2. The operator was advised to keep the cutting device close to these angles and to resect cartilage, cortical, and cancellous bone from the femoral head with typical machining movements. An indicator marking the initial orientation of the bur was provided to support the operator during bone machining. The femoral head was machined for approximately five minutes.

An external irrigation tube was arranged above the cutting head as specified by the manufacturer and connected to a peristaltic pump (disposable irrigation cassette, Stryker, Kalamazoo, MI, USA). The suction end of the disposable irrigation cassette was connected to a gravity infusion bottle filled with the irrigation solution. Per factory-coding of the high-speed drilling system, the irrigation rate was set to 14 ml/min.

The irrigation solution was contaminated with *Staphylococcus aureus* (American Type Culture Collection [ATCC] 29,213) as a tracer for contamination load. It is assumed that the local bacterial count is proportional to the local amount of contamination and that these bacteria, with sizes of ~ 0.5 to 1.5 μm, adhere to the aerosol and particles spread in the OR during bone machining [15–17, 28].

*Staphylococcus aureus* was grown in tryptic soy broth (Merck, Darmstadt, Germany) under aerobic conditions at 37 °C for 24 h. The final concentration of colony-forming units (CFU) of *Staphylococcus aureus* in the test solution was obtained by a colony count and McFarland standardization. The final concentration of the irrigation solution reached 2 × 10^6^ CFU/ml or McFarland 0.5 of staphylococci.

Three test runs were carried out with a total of 12 phantom snippets for optical analysis. The contamination load analysis for CFU counting was done on 12 phantom snippets, 12 room reference snippets, and on three clean snippets which were used as a negative control for each test run. For each test run, the phantom snippets, the room reference snippets, the negative control snippet, and the femoral head were replaced by new ones.

### Contamination load assessment

The phantom snippets from locations A to D and the room reference snippets were subjected to a contamination assessment. To this end, they were harvested from the phantom object with caution by using sterile scissors and tweezers. The Petri dishes (Ø 90 mm) were labeled according to the snippet position and the test run number. The room reference and phantom snippets as well as the negative control snippet were spread out on Columbia blood agar (Becton Dickinson, Franklin Lakes, NJ, USA) and incubated at 37 °C for 24 h (Microbiologic Incubator, IncuLine, VWR International LLC, Darmstadt, Germany).

After incubation, a CFU count *n* was manually determined for each snippet. The CFU counts of the four room reference snippets were averaged to a single value *n*_RR_. Due to the constant irrigation rate, the spent volume of the irrigation solution grows linearly with the machining time *t*_m_. It is assumed that the CFU counts *n* and *n*_RR_ grow linearly with the volumes of the consumed coolant and of the resected tissue. Given these assumptions, a standardized reference-corrected CFU count *n*_s_ for a standard machining time *t*_s_ was introduced:

$${n}_{\mathrm{s}}=\left(n-{n}_{\mathrm{RR}}\right)\frac{{t}_{\mathrm{s}}}{{t}_{\mathrm{m}}}.$$

The standardized CFU count *n*_s_ allows for comparisons between measurements with different reference counts *n*_RR_ and machining times *t*_m_. If *n* − *n*_RR_ < 0, we put *n*_s_ ≝ 0, as there cannot be less than 0 CFU on a snippet. A standard machining time of *t*_s_ = 5 min was used.

### Morphology assessment

**Fig. 3:**
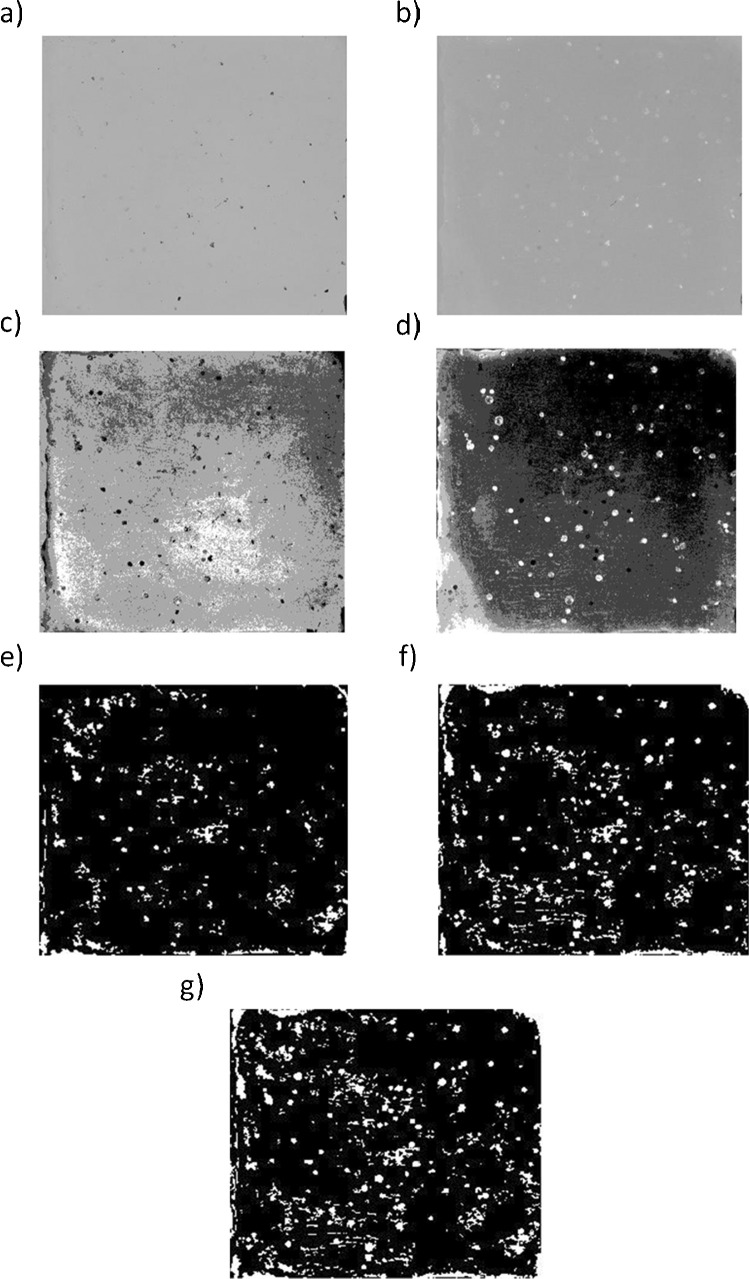
Cropped and rotated photo obtained from a photograph illuminated **a**) with transmission light source and **b**) with reflecting light source. Area clustering to discrimate between particles and background **c**) from transmission light source and **d**) from reflecting light source. Results of the area detection on clustered images **e**) with reflected light source, **f**) with transmission light source, and **g**) the combination of both images

The four phantom snippets from locations 1 to 4 were used for macroscopically morphology assessment to determine the two-dimensional cross section (further referred to as particle size) and the shape of the particles. After each test run, these snippets were collected from the phantom object and placed in sterile empty Petri dishes (Ø 90 mm). The macroscopically morphological sample analysis involved a CCD camera (Canon EOS 60D, 18 Megapixels, sensor size 22.3 × 14.9 mm, pixel dimensions 5184 × 3456, pixel size 4.3 µm) with macro lenses and adapter rings. Exposures were taken from a fixed height above a glass plate with two light sources to minimize shadows. One image was taken with reflecting (ISO 100, 2/5 s f/13 100 mm) and one with transmitting light sources (ISO 100, 1/30 s f/13 100 mm). The pixel edge length was 22 µm, resulting in a pixel area of 484 µm^2^. Both images were rotated and cropped (see Fig. 2a, b). Further, the intensity values were scaled such that 1% of data is saturated at low and high intensities of the image; the image was processed with a median filter. A k-means clustering algorithm was used to detect particles from the background reflectance. From the resulting data image areas containing between 50 and 5000 pixels (see Fig. 3c, d) were selected. All images with the data of different reflection properties were condensed in a single image for reflecting and another image for transmitting light source by adding all areas between each other (see Fig. 3e, f). The resulting two images were combined into one single image on which contaminated areas were detected (see Fig. 3g). The percentage of the areas contaminated was calculated by summing all particle-covered pixels and dividing this number by the total number of image pixels.

While the aerosol particles actually are three-dimensional objects, their contamination capacity is dominated by the two-dimensional cross section of their impact area. To characterize the particle size, we interpret it simplistically as elliptic. Out of this data, the numerical eccentricity *ε* could be calculated of such an ellipse as the ratio of the distance between the foci of the ellipse and its major axis length. An eccentricity of *ε* < 0.25 was classified as a circle, an eccentricity of 0.25 < *ε* < 0.5 was classified as more circular than linear, an eccentricity of 0.5 < *ε* < 0.75 was classified as more linear than circular, and an eccentricity of 0.75 < *ε* < 1 was classified as a linear object.

### Statistical analysis

Descriptive statistics was performed with GraphPad Prism (Version 7, GraphPad Software, Inc., La Jolla, CA, USA) and Excel (Microsoft Office 365 ProPlus, Excel 2016, Microsoft Corporation, Redmond, WA, USA). For each of the 12 snippets evaluated (at locations 1 to 4), the minimum and maximum particle size was determined, as well as its first, second, and third quartiles. Then, the mean and the standard deviation of these five descriptive quantities were calculated. An analogous analysis was added for the CFU counts and for the snippet contamination area (snippets at locations A to D).

## Results

### Contamination assessment

**Fig. 4:**
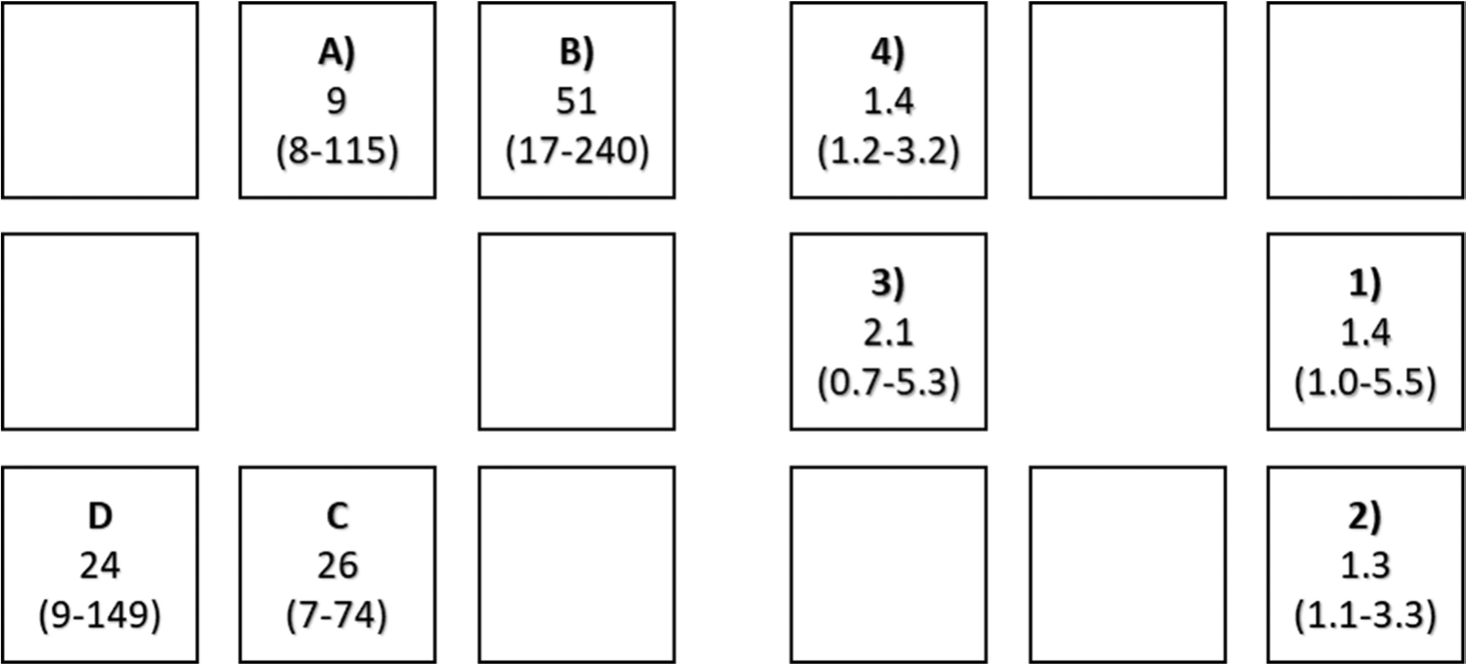
The local contamination load for a standard machining time of 5 min was expressed in terms of the median (range) CFU count **a**) and in terms of the median (range) percentage of area covered by particles **b**)

Across the three test runs, the contamination load analysis showed the highest CFU counts at location B and the lowest CFU counts at location A (see Fig. 4a). The overall median contamination across the 12 phantom snippets (= 3 test runs × 4 snippets) was 25 CFU counts with a range of 7 CFUs to 240 CFUs for the standardized machining time of five minutes. The room reference snippets showed a median contamination of 0 (range 0 to 18) CFU counts.

### Morphological assessment

Based on the optical image analysis, the maximum contamination load of 5.5% was identified at location 3 on the phantom object and the minimum contamination load of 0.7% at location 2 (Fig. 4b). The median percentage of area covered by particles was 1.4 (range: 0.7% to 5.5%).

The particle size ranged from 0.006 mm^2^ (SD 0.009 mm2) to 3.097 mm^2^ (SD 1.428 mm^2^). The median particle size was 0.125 mm^2^ (SD 0.073 mm2) (Table 1).

**Table 1:** Particle size assessment expressed in terms of mean area of particles split by their minimum, maximum, and quartiles. Particles on 12 phantom snippets at locations 1 to 4 were assessed

Area distribution	Mean (standard deviation) area in mm^2^
Minimum	0.006 (0.009)
25% quartile	0.056 (0.032)
Median	0.125 (0.073)
75% quartile	0.262 (0.120)
Maximum	3.097 (1.428)

**Fig. 5:**
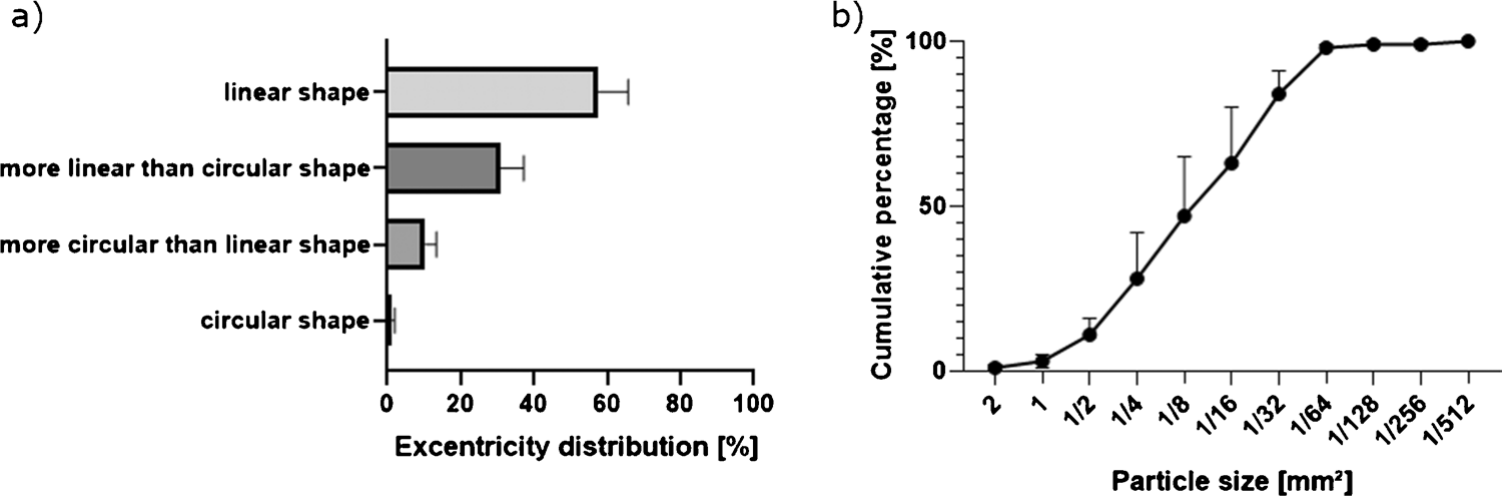
Particle size distribution across 12 phantom snippets expressed in terms of cumulative percentage **a**) and eccentricity distribution across 12 phantom snippets expressed in terms of mean percentage **b**). Bars represent the standard deviation

When considering cumulative percentages, the curve showed an equal distribution of the particle sizes (Fig. 5a).

Figure 5b reports the particle eccentricity. One percent (SD 1%) of the particles had a circular shape (*ε* < 0.25), 10% (SD 3%) were more circular than linear (0.25 < *ε* < 0.5), 31% (SD 6%) showed a more linear than circular shape 0.5 < *ε* < 0.75, and 58% (SD 8%) had a linear shape 0.75 < *ε* < 1. On overall, more than 89% of the particles showed a non-circular shape.

## Discussion

The highest contamination load (highest CFU count) was found on snippet at position B followed by position C and D (see Fig. 4a). These findings are in line with a previous study investigating contamination patterns while machining with a high-speed bur [18]. The authors concluded that besides a “hot spot” of contamination, the detected bacterial load increased with decreasing height and—due to the nature of the rotary burr operation and the parabolic trajectory of particles—accumulated in a centrifugal band [18]. Contamination of surfaces is not only limited to the investigated areas. Nogler et al. showed in two different studies that contaminated areas could be found all over the surgical theater (5 m × 7 m specified in the study) and that the surgeon and the first assistant showed the highest exposure during high-speed burring [15, 16].

When considering the optical image analysis, position 3 was contaminated the strongest (Fig. 4b). This might be due to the specific angulation of the cutting surface of the burr and the resulting spread out of particles in this plane.

Considering a standardized machining time of five minutes, 5% of a snippet area was maximum contaminated. Across 12 phantom snippets, the median contaminated area was 2%. The maximum contamination within the surgical field was observed on snippet at position 3 (distance burr to snippets was 55 cm). This given, it is highly recommend to position equipment not radially to the burring device and not within 55-cm distance, especially when using a navigation camera in computer-aided surgery [29, 30] or other optical systems such as a microscope [31, 32]. Furthermore, burring time should be reduced to a minimum, and cleaning the optical devices might be necessary after the machining process.

The morphology analysis (Fig. 3) showed an equally distributed cumulative percentage curve with particle sizes ranging from 0.006 to 3.097 mm2. The median particle size was 0.125 mm^2^. Due to limitations in the optical measurement, particles smaller than 484 µm^2^ could not be detected; therefore, the study focuses on droplets which settle through gravity. These are particles which can reach by their size the upper respiratory tract and can be filtered by wearing a surgical mask. It is not excluded that smaller particles might be produced during bone resection. They have the potential to reach also the alveoli and provoke infections. The particle eccentricity was non-circular in 89% of the analyzed particles (Fig. 5a). Considering the shape of the particles might be an important aspect when considering filtering capabilities of surgical masks.

Contamination in the surgical setup is not limited to the surgical field but can occur all over the OR when using high-speed burs as assessed in a study by Nogler et al. [15, 16]. In a study of Heinsohn et al. the exposure of the OR personnel by blood aerosols was investigated [33]. The authors conclude that the upper respiratory tract was exposed to aerosolized blood in the OR [33]. Jewett et al. investigated aerosol particles which were generated by power tools such as bone saws, bone drills, or electrocautery in machining and coagulation mode. They concluded that all instruments let to blood-containing aerosol particles smaller than 5 µm (respirable size range) in an OR setting [34]. In a recent study, Hirschmann et al. pointed out that surgical procedures that generate aerosols are at high risk for transmission of viral particles such as SARS-CoV-2, which is known to be present in all body fluids and tissue particles [22].

Surgical masks can be considered as an effective barrier for particles sizes assessed in this study; however, the generation of smaller particles by bone machining cannot be excluded. Therefore, we additionally recommend the usage of sterile helmets in aerosol generating procedures, which is in line with Tokars et al. [35, 36]. The personnel.

should be particularly aware that those helmets might be a contamination source itself, as the control panel might be contaminated over time [20]. Further, gloves should be changed frequently, especially after aerosol generating procedures [21, 37]. Staff members should wear surgical waterproof gaunt, and the number of staff members in the OR should be reduced to a minimum [16]. The personnel present in the OR should be well trained in appropriate safety procedure and should know potential sources of infection [20]. Lastly, patients with known infections should be operated at the end of the day to avoid infection of consecutive patients; careful routine disinfection should be carried out after such procedures [15, 16].

### Limitations

The bone quality of the human femoral heads was not assessed. Machining osteoporotic bones might produce larger particles than dense hard bones. A worst-case scenario has been investigated which differs from a standard surgical setup: All soft tissue was removed around the bone; therefore, no tissue envelope inhibited the spreading of aerosols. No laminar flow was used which would reduce CFU counts within the surgical field [38]. The experiments were carried out in a laboratory setup; in the OR barrier drapes and other equipment might additionally limit spreading of aerosols. The attack angle of the bur was controlled as much as possible by the operator in order to get reproducible results. This might not reflect completely the surgical situation where different burring angles might be necessary depending on the specific surface to be machined.

## Conclusion

It is highly recommended for staff members to avoid areas with high contamination by aerosol generating procedures. Optical systems like navigation cameras or microscopes should not be placed in the contamination hot spot either, as this might compromise their performance. As the filtration efficacy of surgical masks is limited and particles below 0.3 µm might be generated, we suggest surgical helmets be worn by all staff members in the surgical field during aerosol generating procedures. Further, gloves should be changed routinely after the aerosol generating procedures by all staff members present in the surgical field.

## Data Availability

Data is stored on internal servers and handed out on request.
